# Body Weight Dissatisfaction Among Israeli Jewish and Arab Women With Normal or Overweight-Obese Body Mass Index, Israeli INHIS-1, 2003-2004

**Published:** 2009-03-15

**Authors:** Amanda Niskar, Orna Baron-Epel, Noga Garty-Sandalon, Lital Keinan-Boker

**Affiliations:** Office of Preparedness and Emergency Operations, Assistant Secretary for Preparedness and Response, US Department of Health and Human Services. At the time this article was prepared, Dr Niskar was affiliated with Tel Aviv University, Tel Aviv, Israel; University of Haifa, Israel; University of Haifa, Israel; University of Haifa, Israel

## Abstract

**Introduction:**

In Israel, 58.9% of Jewish and Arab Israeli women aged 25 to 64 years are overweight or obese (body mass index ≥25 kg/m^2^). The objective of this analysis is to describe body weight dissatisfaction differences between Jewish and Arab Israeli women with normal or overweight-obese body mass index.

**Methods:**

This analysis included 1,393 Jewish and Arab women who participated in the Israeli National Health Interview Survey, 2003-2004. The survey covered a random sample of the Israeli general population aged 21 years or older. All variables were based on self-report. Body weight dissatisfaction was a multiple-choice question in the survey that offered the following responses: very satisfied, satisfied, reasonably satisfied, not satisfied, or very unsatisfied. Univariate and multivariate analyses were conducted.

**Results:**

Overall, 39.1% of Jewish women reported body weight dissatisfaction, compared with 29.1% of Arab women. Older overweight-obese Arab women had a lower prevalence of body weight dissatisfaction than Jewish women of the same age group, which indicates cultural differences in body weight dissatisfaction among older overweight-obese women. However, cultural differences do not appear to influence body weight dissatisfaction among younger Jewish and Arab women of normal weight.

**Conclusion:**

This study suggests that Jewish and Arab women differ in their perceptions of body weight. Interventions tailored to each group are needed to promote healthy dietary and physical activity behaviors.

## Introduction

More than 400 million adults are estimated to be obese worldwide (1). In Israel, the prevalence of overweight and obesity among Jewish and Arab women aged 25 to 64 years is 58.9% (2). Study samples vary by many factors such as nationality, race, and ethnicity. Thus, results from studies among different populations cannot necessarily be generalized to other populations. A study in Israel among Arab women showed similar findings to studies on US populations regarding an association of increased body mass index (BMI) with onset of type 2 diabetes (3). Despite the public health risks associated with overweight and obesity, we know of no data that describe the relationship between body weight dissatisfaction (BWD), overweight-obese BMI, dieting, and population group among Israeli women. Understanding the possible relationship between BWD, BMI, dieting, and population group among Israeli women is of interest to Israeli health professionals delivering health care services including preventive services to the diverse populations of Israeli women who are in varying levels of acculturation. In addition, findings of this study are relevant to providing health care services to similar populations living in other countries.

Israel is a complex country that is pluralistic, multicultural, and democratic (4). Israel's national health care system is highly developed, and national health insurance covers the entire population. Israel is a small country with approximately 29,000 km^2^ of land (4). The approximately 7.3 million people of Israel originate from more than 30 countries (4). The 2 major ethnic populations are Jews (approximately 80%) and Arabs (approximately 20%), and Israel has 2 official languages, Hebrew and Arabic (4). These 2 populations differ by language, religion, and culture.

Studies describe minority and immigrant populations living in Israel, Australia, and other countries whose weight-related values and behaviors are similar to those of Westernized white women as they become acculturated (5,6). People's food consumption is sensitive to changes in food portion size, the number of people with whom they eat, the amount of food that others eat, and the variety of foods available (7,8). These external environmental factors may explain the finding that body weight often increases or decreases when people move from one area of the world to another, when they enter the college environment, or when they have a change in marital or relationship status (7). Stress-induced eating may be another factor that contributes to the development of obesity (9). Chronic life stress seems to be associated with a greater preference for energy- and nutrient-dense foods high in sugar and fat (9).

Factors associated with BWD include body weight, muscle tone, and body fat (10). Cultural attitudes regarding appearance, physical activity, and health influence BWD (11). In Israel, married women and women with low education are at risk for a sedentary lifestyle, which relates to cultural attitudes about physical activity and may be associated with modernization (12). Identifying factors associated with BWD among Israelis could help health professionals develop tailored interventions to promote healthy weight for Israelis (13). The objective of this analysis is to describe the nationally representative prevalence of BWD among Jewish and Arab Israeli women with normal or overweight-obese BMI, by age, dieting status, education, smoking status, and general health status.

## Methods

### Survey framework

The Ministry of Health's Israel Center for Disease Control (ICDC) conducted the first Israeli National Health Interview Survey (INHIS-1) during 2003 and 2004 (14). Informed consent for each participant was obtained by telephone after a brief explanation about the health survey, including the objectives and importance. People who consented by telephone to participate in the survey were interviewed and were told they could stop the interview at any point and could decline to answer any question at any time. According to Israeli legislation, such a telephone health survey is conducted within the capacity of the ICDC and is not considered medical research. Thus, no approval of an ethical committee was needed for this data collection and analysis.

The INHIS-1 data are nationally representative of the Israeli general population, including the Arab population. INHIS-1 was based on the European Health Interview Survey (EUROHIS) framework initiated in 2000 by the World Health Organization Regional Office for Europe. In 2000 and 2001, EUROHIS field tests were conducted in participating countries, including Israel (15).

### INHIS-1 sample

The study sample consisted of a random sample of the Israeli citizen general population aged 21 years and older. Telephone numbers were selected from a computerized list of national telephone company subscribers estimated to cover 94% of Israeli households, according to the Central Bureau of Statistics (16). The 6% of the population without a national telephone company subscription are likely to have a subscription with another telephone service provider. These other telephone service providers were not accessible in the computerized list used for random selection of survey participants.

### INHIS-1 data collection

The ICDC prepared the EUROHIS questions for application in Israel. The 150 EUROHIS questions were translated into Hebrew and back into English to ensure correct translation. INHIS-1 included 142 EUROHIS questions after 8 questions not relevant to the Israeli population were removed. An additional 129 questions relevant to the Israeli population (eg, regarding population group) or related to specific topics of interest were added to INHIS-1 for a total of 271 questions. The Hebrew questionnaire was then translated into Arabic and Russian and translated back into Hebrew for quality control. The questionnaire was administered over the telephone by trained interviewers from the corresponding population group in Hebrew, Arabic, or Russian. Data were entered and analyzed with a statistical package developed for INHIS-1, and logic checks were performed.

### INHIS-1 women's health module

The findings presented in this analysis are results from the INHIS-1 women's health module (WHM), conducted in 2003. The WHM included the 86 core questions from INHIS-1 and an additional 66 questions relevant to the women of Israel (part of the additional questions described in the above section) to monitor health status and to evaluate the use of health services and health behaviors specific to women. Although modules were not validated, they were pilot tested. For the WHM, 1,491 (27.5%) households could not be located, and 3,927 households from the INHIS-1 were contacted. Households were identified as lost to follow-up after 6 failed attempts to contact the household. The response rate for the WHM was 60.9%. Nonresponses included outright refusals, partially completed interviews, and repeated postponements. Three women did not provide population group data. A total of 1,393 women provided population group data and were available for analysis (1,065 Jewish and 328 Arab). This random sample is nationally representative of the Israeli Jewish and Arab populations.

### Definitions

All variables were based on self-report. Key variables for this analysis were defined on the basis of the survey questions. Population groups were organized into 2 categories: Jewish women or Arab women (Muslim, Christian, and Druze). The survey included other categories of women who did not identify themselves as Jewish Israeli or as Arab Israeli but because their numbers were so small they were designated Jewish Israeli, in accordance with INHIS-1 methods and because these women have similar sociodemographic characteristics to Jewish Israelis.

BWD is a valid assessment commonly used in studies and surveys (17,18). BWD was a multiple-choice question in the survey with 5 possible answers. For this analysis, the 5 categories were grouped into 2 categories: satisfied (0 = very satisfied, satisfied, and reasonably satisfied) or dissatisfied (1 = not satisfied, very unsatisfied). In the survey, women who reported being on a diet were asked to specify 4 diet categories: dieting for weight reduction, dieting for weight maintenance, dieting for health reasons, or dieting for weight gain. For analyses, dieting was grouped into 2 categories: dieting (1 = for weight reduction or weight maintenance) or not dieting (0). Dieting for weight reduction or weight maintenance was the dieting category of interest for this analysis because we were interested in the increasing trends of obesity and overweight among Israeli women. Dieting for weight maintenance is included in the dieting category because the purpose of dieting is to avoid gaining weight and to maintain health.

BMI was calculated as the reported weight without clothes and shoes divided by the square of the reported height without shoes. This analysis is interested in obesity issues and focused on the differences between women of normal BMI and women of overweight or obese BMI. These 2 BMI categories are defined by the World Health Organization as 18.5 kg/m^2 ^to 24.9 kg/m^2 ^(normal BMI) or 25.0 kg/m^2^ or more (overweight-obese BMI) (1). General health status was reported as 1 of 5 categories (good, very good, fair, bad, very bad). For this analysis, general health status was defined in 2 categories as optimal (1 = good, very good) or suboptimal (0 = fair, bad, very bad).

### Statistical analyses

Univariate and multivariate analyses were conducted by using SAS version 9.1 (SAS Institute, Inc, Cary, North Carolina). The effect of population group (Jewish or Arab Israeli women) and BMI on BWD was assessed by age group. Except for the logistic regression modeling, all *P* values were the results of a χ^2^ test. The percentage of women dieting was assessed by BWD, BMI, and population group. To estimate the independent associations of each variable on BWD, χ^2 ^statistics and multiple logistic regression models were conducted with BWD as the dependent variable. Odds ratios and 95% confidence intervals were calculated for each variable, controlling for other variables in the final logistic regression model. Significance was set at a *P* value of <.05. Confounders adjusted for in the final logistic regression model are population group, age, BMI, dieting, marital status, health status, and education.

## Results

Overall demographic characteristics of the Israeli population in 2003 are described in Table 1. Characteristics of the INHIS-1 sample by Jewish and Arab women are presented in Table 2. In this survey, 39.1% of Jewish women and 29.1% of Arab women reported BWD. Among overweight-obese women, 67.6% of Jewish women and 44.9% of Arab women reported BWD. Among overweight-obese women, 45.9% of Jewish women and 39.3% of Arab women with BWD reported dieting for weight reduction or weight maintenance. The percentage of normal-BMI women dieting for weight reduction or weight maintenance was not significantly different between Jewish (20.9%) and Arab (21.4%) women.

Results of the multivariate analyses are presented in Table 3. Smoking, physical activity, and having children were not included in the logistic regression model displayed in Table 3 because these variables were not significantly associated with BWD in either population. Interaction terms between population group, age, BMI, and dieting were not significant in logistic regression modeling.

## Discussion

This is the first analysis describing BWD among Jewish and Arab Israeli women. Differences in the prevalence of BWD were found between these 2 populations. In this INHIS-1 analysis, Arab women were less likely than Jewish women to report BWD. A higher percentage of Arab women than Jewish women were overweight-obese. Another recent Israeli survey that measured body weight found Arab women have a higher rate of obesity than Jewish women (2). Our finding that BWD was associated with an overweight-obese BMI and dieting to lose or maintain weight is consistent with other international research showing that dieting is a common weight-loss strategy among women with BWD (19,20). Chronic dieters are likely to have BWD (11), and older overweight or obese women may have a history of unsuccessful weight loss attempts resulting in depression and BWD (21). A study in South Africa examined the prevalence of BWD in both normal and overweight glucose-intolerant nondiabetic women (18). Both overweight and normal-weight glucose-intolerant women experienced a chronic dieting mindset, binge or uncontrolled eating, and feelings of guilt or depression after a binge in conjunction with BWD (18).

The Israel Heart Fund develops health promotion projects in the community to focus on special populations such as Arab women to prevent and reduce obesity (22).

A main finding of this study is the difference in BWD among young Arab women compared with older Arab women. Muslim Arab people in Israel have a history of less educational attainment than Jewish people in Israel (23). Cohorts born from the mid-1920s to the 1970s experienced a narrowing of population group differences in educational attainment at the lower levels of schooling, but the differences increased at higher levels of education (23).

Our findings that BWD is associated with sociodemographic factors such as population group are consistent with previous studies in America and Europe (20,21). Overweight and obese US minorities were more likely to believe and to self-report that they were not overweight than were nonminorities (24). Targeted media campaigns and culturally tailored community educational events are examples of strategies to raise awareness about healthy living to promote healthy eating and healthy physical activity patterns among US minority populations (25).

Our finding showing that older overweight-obese Arab women reported a lower prevalence of BWD than Jewish women of the same age group may reflect a difference in the degree of urbanization and industrialization between Jewish and Arab Israelis (26). The morbidity and premature mortality seen in the Arab population is higher than that of the Jewish population (27). This finding that Arab overweight-obese women were more likely to be satisfied with their weight is of concern, because their obesity rates place them at higher risk for developing chronic disease. For example, the prevalence of diabetes is higher among Arab Israelis than among Jewish Israelis (27). Approximately two-thirds of Israeli Arabs diagnosed with type 2 diabetes were women, and most of these women had a higher BMI at diagnosis of type 2 diabetes than did men (3).

Although older Arab and Jewish women differed in terms of BWD, our results demonstrated a similar prevalence of BWD among young Jewish and Arab women. The similarities among Arab and Jewish young women's attitudes to body weight may suggest that the Arab community is in transition from a more traditional and collective way of life to a more individualistic way of life, adopting more modern lifestyles (26). Similarly, immigrants who lived in Israel for 4 to 15 years adopted Israeli cultural norms for eating patterns related to obesity and eating disorders (6).

A major strength of this analysis is that the results are nationally representative for adult women in Israel. INHIS-1 is among the largest national surveys in Israel, and this is the first time that BWD was studied in Israel. The BMI calculations in our study are limited by the self-reporting method of gathering height and weight data. Most women underestimate their weight and overestimate their height, resulting in BMI miscalculations (28). The degree of self-report weight inaccuracies can vary by population group (29), and chronic dieters are likely to overestimate their body weight (11). Actual height and weight measurements would be more reliable for studies calculating BMI. Another strength of this analysis is that multivariate analyses were conducted with and without controlling for education, income, and employment, and the same differences were observed, which indicates that cultural issues may explain the differences rather than income or education.

### Conclusion

Israeli Jewish and Arab women differ with regard to BWD. To prevent eating disorders and chronic diseases, Israeli women need to balance healthy behaviors and body weight attitudes. Interventions should be tailored to age group, education level, religion, marital status, language, and cultural traditions to promote satisfaction with normal BMI and to prevent obesity and related chronic diseases. Interventions are needed to promote proper nutrition, regular physical activity, and stress-reduction techniques (9,13). Some evidence suggests that eating is an automatic behavior that is not strongly influenced by nutrition education and that physical activity cannot compete with increased consumption of food (8). Therefore, additional interventions are needed that shape the external environment relevant to person, place, and time of food purchase, preparation, and consumption (7). Public health professionals in other countries with minority and immigrant populations may consider developing interventions to prevent obesity that are tailored to age group, religion, language, and culture of each population group.

## Figures and Tables

**Table 1:** Characteristics of Jews, Muslim Arabs, Christian Arabs, and Druze in Israel, 2003

Characteristic	Value			

	Jews	Arabs		

		**Christian**	**Druze**	**Muslim**
Population	5,165,400	115,700	110,800	1,072,500
Population younger than 15 y, %	25.5	28.5	34.8	41.2
Full high school education with matriculation certificate, %	56.3	67.4	48.3	49.4
Employed, ages 15-64 y, %	55.0	50.8	35.5	35.9
Urbanization: living in towns^a^, %	90.8	99.1	97.4	92.7
Of these, living in towns with <50,000 inhabitants, %	32.7	53.7	96.7	63.8
Annual population growth, %	3.8	1.9	3.8	4.4
Total fertility rate^b^	2.7	2.4	2.9	4.5
Infant mortality rate (per 1,000 liveborn)	3.6	3.2	7.1	8.8

a Urban settlements are defined as those with at least 2,000 inhabitants.

b Average number of children per woman during her lifetime.

**Table 2:** Characteristics of Israeli Women Who Participated in the First Israeli National Health Interview Survey, 2003 (N = 1,393)

**Characteristic**		Jewish Women, n (%)	Arab Women, n (%)	*P* Value^a^
Total		1,065 (76.4)	328 (23.6)	NA
Age group, y	21-34	304 (28.5)	128 (39.0)	.007
	35-44	199 (18.7)	60 (18.3)	
	45-54	230 (21.6)	55 (16.8)	
	55-64	167 (15.7)	45 (13.7)	
	≥65	165 (15.5)	40 (12.2)	
Education, y	<12	150 (14.2)	189 (57.8)	<.001
	12	561 (53.0)	108 (33.0)	
	>12	348 (32.9)	30 (9.2)	
Marital status	Married	767 (72.2)	246 (75.0)	.31
	Not married	296 (27.8)	82 (25.0)	
Net monthly income, NIS^b^	<5,200	137 (18.2)	125 (56.3)	<.001
	5,200-8,500	139 (18.5)	55 (24.8)	
	>8,500	476 (63.3)	42 (18.9)	
Employment	Yes	593 (58.7)	59 (18.9)	<.001
	No	417 (41.3)	254 (81.1)	
Smoking^c^	Yes	217 (20.4)	21 (6.4)	<.001
	No	848 (79.6)	307 (93.6)	
General health status^d^	Suboptimal	160 (34.0)	96 (35.4)	.70
	Optimal	310 (66.0)	175 (64.6)	
Body mass index^e^	Normal	525 (55.7)	101 (44.1)	.002
	Overweight-obese	417 (44.3)	128 (55.9)	
Dieting to lose or maintain weight	Yes	308 (29.0)	63 (19.5)	<.001
	No	754 (71.0)	260 (80.5)	

Abbreviation: NA, not applicable; NIS, New Israeli Shekel.

a *P* values were derived from χ^2^ tests.

b Net monthly income was described in terms of the NIS.

c Defined as current smoker or nonsmoker.

d General health status was defined as optimal (good, very good) or suboptimal (fair, bad, very bad).

e Body mass index was defined as normal (18.5-24.9 kg/m^2^) and overweight-obese (≥25.0 kg/m^2^).

**Table 3:** Odds of Factors Influencing Body Weight Dissatisfaction Among Israeli Women (n = 626^a^), First Israeli National Health Interview Survey, 2003

**Variables**	**OR (95% CI)**	***P* value^b^**
Jewish women [reference] vs Arab women	2.19 (1.29-3.71)	.004
Age [continuous from young to old]	0.97 (0.95-0.98)	<.001
Body mass index [continuous from low to high]	1.39 (1.31-1.48)	<.001
Dieting [reference] vs non-dieting	2.44 (1.55-3.84)	<.001
Married [reference] vs single	1.63 (0.99-2.82)	.06
Health status: optimal [reference] vs suboptimal	0.43 (0.26-0.70)	<.001
Education: <12 years [reference] vs ≤12 years	1.35 (0.98-1.87)	.06

Abbreviation: OR, odds ratio; CI, confidence interval.

a Only participants with information regarding all variables were included in the logistic regression modeling analyses.

b *P* values were derived from multiple logistic regression.
